# A hydrogen sulfate salt of chlordiazepoxide

**DOI:** 10.1107/S1600536812024920

**Published:** 2012-06-13

**Authors:** Veronica Diesen, Cláudio Lousada, Andreas Fischer

**Affiliations:** a Division of Applied Physical Chemistry, School of Chemical Science and Engineering, Royal Institute of Technology (KTH), 100 44 Stockholm, Sweden

## Abstract

Crystals of the hydrogen sulfate salt of chlordiazepoxide (systematic name: 7-chloro-*N*-methyl-5-phenyl-2,3-dihydro-1*H*-1,4-benzodiazepin-2-iminium 4-oxide hydrogen sulfate), C_16_H_15_ClN_3_O^+^·HSO_4_^−^, were obtained from a solution of chlordiazepoxide and sulfuric acid in methanol. The structure features chlordiazepoxide mol­ecules that are protonated at the imine N atom. The seven-membered ring adopts a boat conformation with the CH_2_ group as the prow and the two aryl C atoms as the stern. The dihedral angle between the benzene rings is 72.41 (6)°. In the crystal, the HSO_4_^−^ anion acts as a bridging group between two chlordiazepoxide cations. The H atom of the protonated imino N forms an N—H⋯O hydrogen bond with a hydrogen sulfate ion. The anion in turn forms two hydrogen bonds, O—H⋯O with the anion as donor and N—H⋯O with the anion as acceptor, to generate an *R*_2_^2^(10) loop. Each HSO_4_^−^ anion connects two chlordiazepoxide moieties of the same chirality.

## Related literature

For general background to benzodiazepines, the structures of two polymorphs of chlordiazepoxide and a chlordiazepoxide dichloro­methane solvate, see: Fischer (2012 ▶) and references therein. For the structure of chlordiazepoxide hydro­chloride, see: Herrnstadt *et al.* (1979 ▶). For the synthesis of chlordiazepoxide, see: Sternbach *et al.* (1961 ▶). For acid–base equlibria of chlordiazepoxide and related compounds, see: Yang (1995 ▶). For the graph-set motifs, see: Etter *et al.* (1990 ▶).
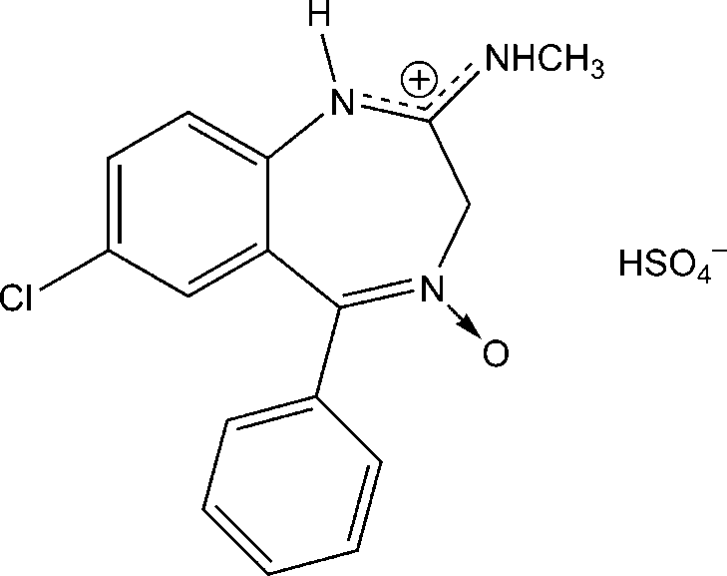


## Experimental

### 

#### Crystal data

C_16_H_15_ClN_3_O^+^·HSO_4_^−^
*M* *_r_* = 397.84
Monoclinic, 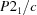

*a* = 13.9899 (6) Å
*b* = 8.7579 (10) Å
*c* = 13.9084 (6) Å
β = 99.657 (9)°
*V* = 1679.9 (2) Å^3^
*Z* = 4
Mo *K*α radiation
μ = 0.39 mm^−1^
*T* = 173 K
0.58 × 0.54 × 0.14 mm

#### Data collection

Bruker–Nonius KappaCCD diffractometer
Absorption correction: multi-scan (*SADABS*; Sheldrick, 2003 ▶) *T*_min_ = 0.806, *T*_max_ = 0.947
23827 measured reflections
3835 independent reflections
2802 reflections with *I* > 2σ(*I*)
*R*_int_ = 0.052

#### Refinement

*R*[*F* ^2^ > 2σ(*F* ^2^)] = 0.037
*wR*(*F* ^2^) = 0.088
*S* = 1.03
3835 reflections
245 parameters
1 restraint
H atoms treated by a mixture of independent and constrained refinement
Δρ_max_ = 0.33 e Å^−3^
Δρ_min_ = −0.39 e Å^−3^

### 

Data collection: *COLLECT* (Nonius, 1999 ▶); cell refinement: *DIRAX* (Duisenberg, 1992 ▶); data reduction: *EVALCCD* (Duisenberg *et al.*, 2003 ▶); program(s) used to solve structure: *SHELXS97* (Sheldrick, 2008 ▶); program(s) used to refine structure: *SHELXL97* (Sheldrick, 2008 ▶); molecular graphics: *DIAMOND* (Brandenburg, 2007 ▶); software used to prepare material for publication: *publCIF* (Westrip, 2010 ▶).

## Supplementary Material

Crystal structure: contains datablock(s) global, I. DOI: 10.1107/S1600536812024920/hb6784sup1.cif

Structure factors: contains datablock(s) I. DOI: 10.1107/S1600536812024920/hb6784Isup2.hkl

Supplementary material file. DOI: 10.1107/S1600536812024920/hb6784Isup3.cml

Additional supplementary materials:  crystallographic information; 3D view; checkCIF report

## Figures and Tables

**Table 1:** Hydrogen-bond geometry (Å, °)

*D*—H⋯*A*	*D*—H	H⋯*A*	*D*⋯*A*	*D*—H⋯*A*
N3—H3*A*⋯O5	0.82 (2)	1.95 (2)	2.764 (2)	171 (2)
N1—H1⋯O3^i^	0.84 (2)	1.93 (2)	2.741 (2)	162 (2)
O2—H2*A*⋯O1	0.81 (3)	1.78 (3)	2.583 (2)	170 (3)

Symmetry code: (i) 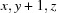.

**Fig. 1.**
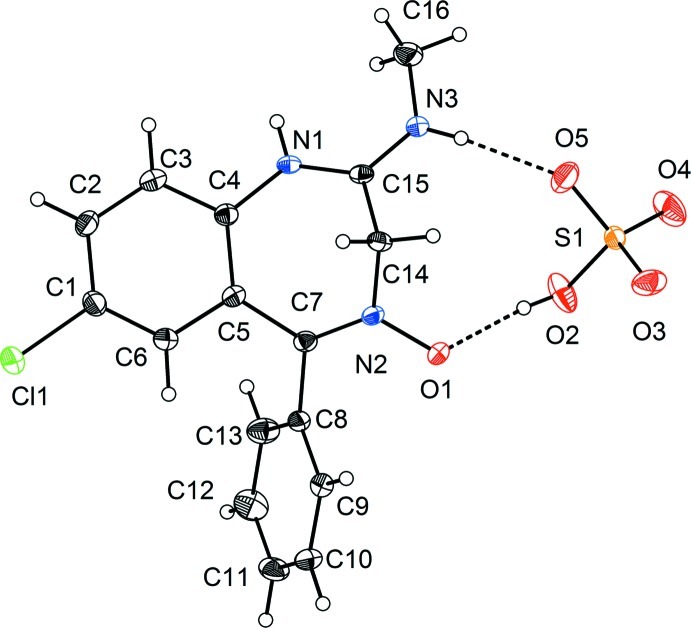
The molecular structure of the title compound. Displacement ellipsoids are drawn at the 50% probability level. Hydrogen bonds shown as dashed lines.

**Fig. 2.**
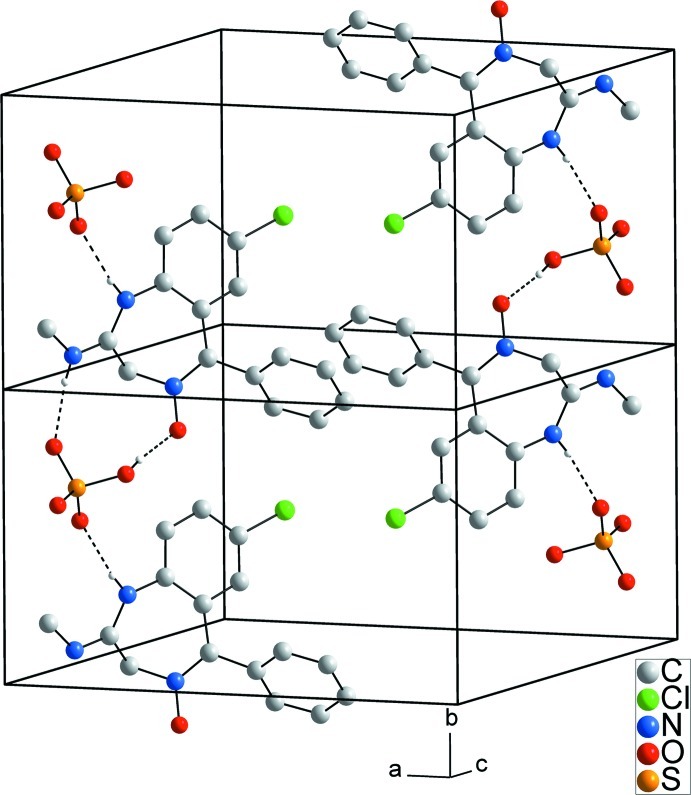
The chains formed by hydrogen bonding between chlordiazepoxide cations and hydrogen sulfate anions. The figure shows two unit cells. H atoms (except those involved in H bonds) are omitted. Hydrogen bonds shown as dashed lines.

## supplementary crystallographic information

### Comment

Since chlordiazepoxide first was released into the market, benzodiazepines have become the most important pharmaceutical compounds being used as anxiolytics, hypnotics and anti-convulsants. However, the knowledge of benzodiazepine salts is still limited.

Chlordiazepoxide can be easily protonated and the protonation appears to occur on the imine nitrogen atom, which could be shown both by the structure determination of the hydrochloride (Herrnstadt *et al.* 1979) and by solution studies (Yang, 1995). In chlordiazepoxide, the dichloromethane solvate and the hydrochloride, dimers of chlordiazepoxide moieties are observed. In order to study the influence of the counterion on the hydrogen bonding pattern, we crystallized chlordiazepoxide hydrogen sulfate from a methanol solution.

The structure of the title compound features chlordiazepoxide molecules, that are protonated at N1 (Fig. 1). The seven-membered ring adopts a boat conformation with the CH_2_ group as the prow and the two aromatic C atoms as the stern. The HSO_4_^-^ acts as a bridging group between two chlordiazepoxide cations. The hydrogen atom of the protonated imino-N forms a N–H···O bond with a hydrogen sulfate ion. The anion forms in turn two hydrogen bonds, one O–H···O group where the anion acts as donor and one N–H···O group where it acts as acceptor. These three H bonds yield a *R*^2^_2_(10) loop. Each HSO_4_^-^ group connects two chlordiazepoxide moieties of the same chirality. Thus, each hydrogen sulfate group acts as a bridging group, linking two chlordiazepoxide moieties yielding infinite chains (Fig. 2). Each chain contains only one enantiomer of the molecule. The dihedral angle between the benzene rings is 72.41 (6)°.

### Experimental

Chlordiazepoxide was synthesized according to the procedure described by Sternbach *et al.* (1961). Crystals of the title compound were obtained as hexagonal, yellow plates by slow evaporation of a solution of 25 mg chlordiazepoxide and 7.7 mg sulfuric acid (95%) in 5 ml of methanol at room temperature.

### Refinement

C–H hydrogen atoms were placed at calculated positions and refined riding on the respective carrier atom with *U*_iso_=1.2*U*_eq_ of the carrier atom (1.5 *U*_eq_ for the methyl group) and with d(C–H)=0.95Å for aromatic H atoms, 0.99Å for methylene-H atoms and 0.98Å for methyl-H atoms. The torsion angle between the NH group the methyl group in the NHCH_3_ side chain was refined as well. O–H and N–H hydrogen atoms were located from the difference-Fourier map and the respective bond lengths were refined. The interatomic distance N1–H1 was restrained to 0.88 (2) Å because free refinement did not yield a satisfactory result.

### Crystal data

**Table:**

C_16_H_15_ClN_3_O^+^·HSO_4_^−^	*F*(000) = 824
*M**_r_* = 397.84	*D*_x_ = 1.573 Mg m^−^^3^
Monoclinic, *P*2_1_/*c*	Mo *K*α radiation, λ = 0.71073 Å
Hall symbol: -P 2ybc	Cell parameters from 84 reflections
*a* = 13.9899 (6) Å	θ = 4.2–20.7°
*b* = 8.7579 (10) Å	µ = 0.39 mm^−^^1^
*c* = 13.9084 (6) Å	*T* = 173 K
β = 99.657 (9)°	Plate, yellow
*V* = 1679.9 (2) Å^3^	0.58 × 0.54 × 0.14 mm
*Z* = 4	

### Data collection

**Table:**

Bruker–Nonius KappaCCD diffractometer	2802 reflections with *I* > 2σ(*I*)
Radiation source: fine-focus sealed tube	*R*_int_ = 0.052
φ and ω scans	θ_max_ = 27.5°, θ_min_ = 4.5°
Absorption correction: multi-scan (*SADABS*; Sheldrick, 2003)	*h* = −18→17
*T*_min_ = 0.806, *T*_max_ = 0.947	*k* = −11→11
23827 measured reflections	*l* = −18→18
3835 independent reflections	

### Refinement

**Table:**

Refinement on *F*^2^	Primary atom site location: structure-invariant direct methods
Least-squares matrix: full	Secondary atom site location: difference Fourier map
*R*[*F*^2^ > 2σ(*F*^2^)] = 0.037	Hydrogen site location: inferred from neighbouring sites
*wR*(*F*^2^) = 0.088	H atoms treated by a mixture of independent and constrained refinement
*S* = 1.03	*w* = 1/[σ^2^(*F*_o_^2^) + (0.0365*P*)^2^ + 1.1198*P*] where *P* = (*F*_o_^2^ + 2*F*_c_^2^)/3
3835 reflections	(Δ/σ)_max_ < 0.001
245 parameters	Δρ_max_ = 0.33 e Å^−^^3^
1 restraint	Δρ_min_ = −0.39 e Å^−^^3^
13 constraints	

### Special details

**Table:**

Geometry. All e.s.d.'s (except the e.s.d. in the dihedral angle between two l.s. planes) are estimated using the full covariance matrix. The cell e.s.d.'s are taken into account individually in the estimation of e.s.d.'s in distances, angles and torsion angles; correlations between e.s.d.'s in cell parameters are only used when they are defined by crystal symmetry. An approximate (isotropic) treatment of cell e.s.d.'s is used for estimating e.s.d.'s involving l.s. planes.
Refinement. Refinement of *F*^2^ against ALL reflections. The weighted *R*-factor *wR* and goodness of fit *S* are based on *F*^2^, conventional *R*-factors *R* are based on *F*, with *F* set to zero for negative *F*^2^. The threshold expression of *F*^2^ > σ(*F*^2^) is used only for calculating *R*-factors(gt) *etc*. and is not relevant to the choice of reflections for refinement. *R*-factors based on *F*^2^ are statistically about twice as large as those based on *F*, and *R*- factors based on ALL data will be even larger.

### Fractional atomic coordinates and isotropic or equivalent isotropic displacement parameters (Å^2^)

**Table:**

	*x*	*y*	*z*	*U*_iso_*/*U*_eq_	
C1	0.29397 (13)	0.9226 (2)	0.03883 (14)	0.0192 (4)	
C2	0.23368 (14)	1.0175 (2)	0.08120 (14)	0.0196 (4)	
C3	0.18881 (14)	0.9588 (2)	0.15418 (14)	0.0189 (4)	
C4	0.20316 (13)	0.8080 (2)	0.18584 (13)	0.0154 (4)	
C5	0.26573 (13)	0.7120 (2)	0.14382 (13)	0.0157 (4)	
C6	0.31045 (13)	0.7742 (2)	0.06926 (14)	0.0185 (4)	
C7	0.29408 (13)	0.5551 (2)	0.17423 (13)	0.0147 (4)	
C8	0.39263 (13)	0.5004 (2)	0.16413 (13)	0.0163 (4)	
C9	0.40329 (14)	0.3805 (2)	0.10173 (14)	0.0192 (4)	
C10	0.49545 (14)	0.3410 (2)	0.08574 (15)	0.0233 (4)	
C11	0.57571 (14)	0.4185 (3)	0.13293 (15)	0.0253 (5)	
C12	0.56519 (15)	0.5383 (3)	0.19530 (16)	0.0288 (5)	
C13	0.47339 (14)	0.5800 (3)	0.21034 (15)	0.0251 (5)	
C14	0.13111 (13)	0.5056 (2)	0.20202 (13)	0.0161 (4)	
C15	0.12400 (12)	0.6247 (2)	0.27772 (13)	0.0144 (4)	
C16	0.06161 (16)	0.6949 (3)	0.42636 (15)	0.0252 (5)	
Cl1	0.35131 (4)	0.99179 (6)	−0.05429 (4)	0.02899 (14)	
N1	0.15794 (11)	0.76324 (19)	0.26510 (11)	0.0159 (3)	
N2	0.23277 (11)	0.46083 (18)	0.20355 (11)	0.0149 (3)	
N3	0.08275 (12)	0.5887 (2)	0.35181 (12)	0.0173 (3)	
O1	0.25496 (9)	0.32031 (15)	0.23206 (10)	0.0211 (3)	
O2	0.21788 (10)	0.2042 (2)	0.39254 (12)	0.0328 (4)	
O3	0.08836 (10)	0.02501 (17)	0.33641 (11)	0.0304 (4)	
O4	0.12080 (11)	0.1162 (2)	0.50292 (11)	0.0361 (4)	
O5	0.04859 (11)	0.28024 (17)	0.37061 (12)	0.0309 (4)	
S1	0.11383 (3)	0.15276 (5)	0.40179 (3)	0.01669 (12)	
H2	0.2234	1.1204	0.0605	0.024*	
H3	0.1471	1.0227	0.1837	0.023*	
H6	0.3529	0.7123	0.0393	0.022*	
H9	0.3481	0.3259	0.0702	0.023*	
H10	0.5033	0.2602	0.0421	0.028*	
H11	0.6386	0.3895	0.1225	0.030*	
H12	0.6206	0.5915	0.2275	0.035*	
H13	0.4656	0.6631	0.2523	0.030*	
H14A	0.0928	0.4149	0.2145	0.019*	
H14B	0.1037	0.5461	0.1368	0.019*	
H16A	0.1194	0.7562	0.4500	0.038*	
H16B	0.0429	0.6375	0.4808	0.038*	
H16C	0.0083	0.7625	0.3983	0.038*	
H1	0.1412 (14)	0.834 (2)	0.2988 (14)	0.019*	
H3A	0.0662 (16)	0.499 (3)	0.3548 (16)	0.021*	
H2A	0.2223 (18)	0.242 (3)	0.340 (2)	0.039*	

### Atomic displacement parameters (Å^2^)

**Table:**

	*U*^11^	*U*^22^	*U*^33^	*U*^12^	*U*^13^	*U*^23^
C1	0.0178 (9)	0.0210 (11)	0.0193 (10)	−0.0002 (8)	0.0049 (7)	0.0032 (8)
C2	0.0213 (10)	0.0141 (10)	0.0233 (10)	0.0017 (8)	0.0030 (8)	0.0017 (8)
C3	0.0186 (10)	0.0169 (10)	0.0212 (10)	0.0034 (8)	0.0035 (7)	−0.0027 (8)
C4	0.0146 (9)	0.0157 (10)	0.0157 (9)	0.0002 (7)	0.0023 (7)	−0.0015 (8)
C5	0.0159 (9)	0.0139 (10)	0.0171 (9)	0.0013 (7)	0.0025 (7)	−0.0013 (8)
C6	0.0181 (9)	0.0186 (11)	0.0197 (10)	0.0026 (8)	0.0061 (7)	0.0007 (8)
C7	0.0167 (9)	0.0152 (10)	0.0124 (9)	0.0013 (7)	0.0029 (7)	−0.0020 (7)
C8	0.0157 (9)	0.0164 (10)	0.0178 (9)	0.0042 (8)	0.0056 (7)	0.0035 (8)
C9	0.0192 (10)	0.0170 (11)	0.0223 (10)	0.0011 (8)	0.0065 (8)	0.0017 (8)
C10	0.0261 (11)	0.0204 (11)	0.0258 (11)	0.0076 (9)	0.0109 (8)	0.0006 (9)
C11	0.0168 (10)	0.0330 (13)	0.0273 (11)	0.0091 (9)	0.0076 (8)	0.0065 (10)
C12	0.0162 (10)	0.0372 (14)	0.0318 (12)	−0.0031 (9)	0.0001 (8)	−0.0023 (10)
C13	0.0224 (11)	0.0259 (12)	0.0270 (11)	−0.0004 (9)	0.0044 (8)	−0.0089 (9)
C14	0.0136 (9)	0.0176 (10)	0.0178 (9)	0.0003 (7)	0.0052 (7)	−0.0031 (8)
C15	0.0110 (8)	0.0165 (10)	0.0159 (9)	0.0035 (7)	0.0023 (7)	−0.0004 (8)
C16	0.0315 (11)	0.0263 (12)	0.0201 (10)	0.0063 (9)	0.0113 (8)	0.0000 (9)
Cl1	0.0333 (3)	0.0255 (3)	0.0325 (3)	0.0051 (2)	0.0179 (2)	0.0108 (2)
N1	0.0191 (8)	0.0146 (9)	0.0156 (8)	0.0026 (7)	0.0073 (6)	−0.0036 (7)
N2	0.0178 (8)	0.0125 (8)	0.0151 (8)	0.0032 (6)	0.0048 (6)	−0.0009 (6)
N3	0.0202 (8)	0.0142 (9)	0.0191 (8)	0.0019 (7)	0.0082 (6)	−0.0007 (7)
O1	0.0272 (7)	0.0124 (7)	0.0265 (8)	0.0044 (6)	0.0123 (6)	0.0029 (6)
O2	0.0210 (8)	0.0484 (11)	0.0283 (8)	−0.0117 (7)	0.0018 (6)	0.0130 (8)
O3	0.0296 (8)	0.0227 (8)	0.0402 (9)	−0.0017 (6)	0.0100 (7)	−0.0145 (7)
O4	0.0409 (9)	0.0502 (11)	0.0195 (8)	0.0005 (8)	0.0113 (6)	0.0093 (7)
O5	0.0319 (8)	0.0177 (8)	0.0434 (9)	0.0045 (6)	0.0071 (7)	0.0043 (7)
S1	0.0206 (2)	0.0146 (2)	0.0162 (2)	−0.00200 (19)	0.00691 (17)	−0.00031 (19)

### Geometric parameters (Å, º)

**Table:**

C1—C6	1.375 (3)	N2—O1	1.314 (2)
C1—C2	1.384 (3)	O2—S1	1.5495 (15)
C1—Cl1	1.7426 (19)	O3—S1	1.4481 (15)
C2—C3	1.379 (3)	O4—S1	1.4299 (15)
C3—C4	1.396 (3)	O5—S1	1.4611 (15)
C4—C5	1.409 (3)	C2—H2	0.9500
C4—N1	1.415 (2)	C3—H3	0.9500
C5—C6	1.407 (3)	C6—H6	0.9500
C5—C7	1.473 (3)	C9—H9	0.9500
C7—N2	1.304 (2)	C10—H10	0.9500
C7—C8	1.488 (2)	C11—H11	0.9500
C8—C9	1.386 (3)	C12—H12	0.9500
C8—C13	1.390 (3)	C13—H13	0.9500
C9—C10	1.388 (3)	C14—H14A	0.9900
C10—C11	1.381 (3)	C14—H14B	0.9900
C11—C12	1.385 (3)	C16—H16A	0.9800
C12—C13	1.384 (3)	C16—H16B	0.9800
C14—N2	1.472 (2)	C16—H16C	0.9800
C14—C15	1.497 (3)	N1—H1	0.837 (15)
C15—N3	1.301 (2)	N3—H3A	0.82 (2)
C15—N1	1.325 (2)	O2—H2A	0.81 (3)
C16—N3	1.460 (3)		
			
C6—C1—C2	121.28 (18)	O4—S1—O2	103.70 (9)
C6—C1—Cl1	118.89 (15)	O3—S1—O2	107.94 (9)
C2—C1—Cl1	119.83 (16)	O5—S1—O2	107.58 (9)
C3—C2—C1	118.37 (18)	C3—C2—H2	120.8
C2—C3—C4	121.68 (18)	C1—C2—H2	120.8
C3—C4—C5	120.00 (17)	C2—C3—H3	119.2
C3—C4—N1	116.63 (16)	C4—C3—H3	119.2
C5—C4—N1	123.22 (17)	C1—C6—H6	119.3
C6—C5—C4	117.30 (17)	C5—C6—H6	119.3
C6—C5—C7	116.17 (16)	C8—C9—H9	120.4
C4—C5—C7	126.42 (17)	C10—C9—H9	120.4
C1—C6—C5	121.36 (18)	C11—C10—H10	119.9
N2—C7—C5	121.40 (16)	C9—C10—H10	119.9
N2—C7—C8	119.59 (17)	C10—C11—H11	119.8
C5—C7—C8	118.85 (16)	C12—C11—H11	119.8
C9—C8—C13	120.34 (17)	C13—C12—H12	120.2
C9—C8—C7	120.11 (17)	C11—C12—H12	120.2
C13—C8—C7	119.25 (17)	C12—C13—H13	120.0
C8—C9—C10	119.30 (18)	C8—C13—H13	120.0
C11—C10—C9	120.29 (19)	N2—C14—H14A	109.5
C10—C11—C12	120.47 (18)	C15—C14—H14A	109.5
C13—C12—C11	119.6 (2)	N2—C14—H14B	109.5
C12—C13—C8	120.01 (19)	C15—C14—H14B	109.5
N2—C14—C15	110.70 (15)	H14A—C14—H14B	108.1
N3—C15—N1	122.88 (17)	N3—C16—H16A	109.5
N3—C15—C14	118.61 (17)	N3—C16—H16B	109.5
N1—C15—C14	118.50 (16)	H16A—C16—H16B	109.5
C15—N1—C4	125.00 (16)	N3—C16—H16C	109.5
C7—N2—O1	123.56 (15)	H16A—C16—H16C	109.5
C7—N2—C14	120.75 (16)	H16B—C16—H16C	109.5
O1—N2—C14	115.63 (14)	C15—N1—H1	117.9 (15)
C15—N3—C16	125.30 (18)	C4—N1—H1	115.7 (15)
O4—S1—O3	114.42 (10)	C15—N3—H3A	116.0 (16)
O4—S1—O5	113.54 (10)	C16—N3—H3A	118.7 (16)
O3—S1—O5	109.17 (9)	S1—O2—H2A	114.0 (18)

### Hydrogen-bond geometry (Å, º)

**Table:**

*D*—H···*A*	*D*—H	H···*A*	*D*···*A*	*D*—H···*A*
N3—H3*A*···O5	0.82 (2)	1.95 (2)	2.764 (2)	171 (2)
N1—H1···O3^i^	0.84 (2)	1.93 (2)	2.741 (2)	162 (2)
O2—H2*A*···O1	0.81 (3)	1.78 (3)	2.583 (2)	170 (3)

Symmetry code: (i) *x*, *y*+1, *z*.
